# STRAD pseudokinases regulate axogenesis and LKB1 stability

**DOI:** 10.1186/1749-8104-9-5

**Published:** 2014-03-04

**Authors:** Biliana O Veleva-Rotse, James L Smart, Annette F Baas, Benjamin Edmonds, Zi-ming Zhao, Allyson Brown, Lillian R Klug, Kelly Hansen, Gabrielle Reilly, Alexandria P Gardner, Krishnaveni Subbiah, Eric A Gaucher, Hans Clevers, Anthony P Barnes

**Affiliations:** 1 Department of Pediatrics-Doernbecher, Children’s Hospital, Portland, OR 97239, USA; 2 Neuroscience Graduate Program, Oregon Health and Science University, Portland, OR 97239, USA; 3 Hubrecht Institute, Utrecht University, Utrecht, The Netherlands; 4 School of Biology, Georgia Institute of Technology, Atlanta, GA, USA; 5 George Fox University, Newberg, OR, USA; 6 Department of Cell and Developmental Biology, Oregon Health and Science University, Portland, OR 97239, USA; 7 Oregon Health and Science University, 3181 SW Sam Jackson Pk Rd, Mailcode: L481, Portland, OR 97239, USA

**Keywords:** LKB1, Neurodevelopment, Axon, Pseudokinase, STRAD

## Abstract

**Background:**

Neuronal polarization is an essential step of morphogenesis and connectivity in the developing brain. The serine/threonine kinase LKB1 is a key regulator of cell polarity, metabolism, tumorigenesis, and is required for axon formation. It is allosterically regulated by two related and evolutionarily conserved pseudokinases, STe20-Related ADapters (STRADs) α and β. The roles of STRADα and STRADβ in the developing nervous system are not fully defined, nor is it known whether they serve distinct functions.

**Results:**

We find that STRADα is highly spliced and appears to be the primal STRAD paralog. We report that each STRAD is sufficient for axogenesis and promoting cell survival in the developing cortex. We also reveal a reciprocal protein-stabilizing relationship in vivo between LKB1 and STRADα, whereby STRADα specifically maintains LKB1 protein levels via cytoplasmic compartmentalization.

**Conclusions:**

We demonstrate a novel role for STRADβ in axogenesis and also show for the first time in vivo that STRADα, but not STRADβ, is responsible for LKB1 protein stability.

## Background

**Figure 1:**
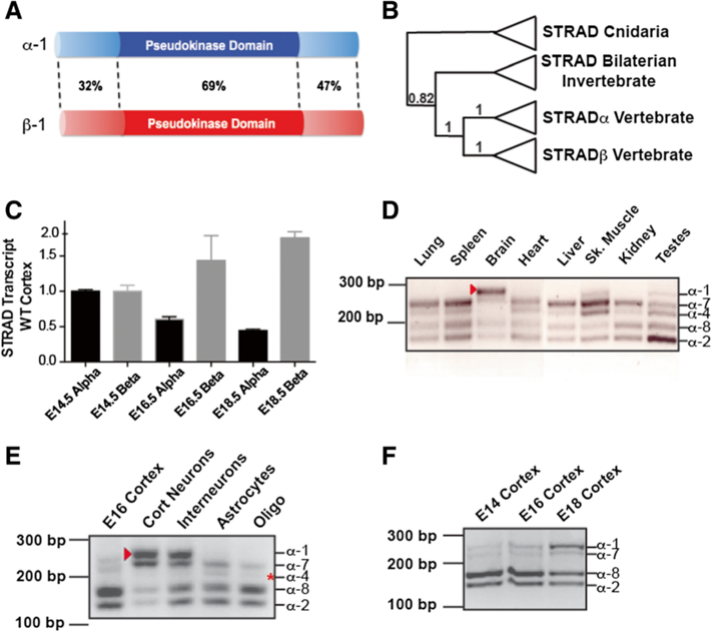
**STRAD splice forms are expressed in a tissue-specific manner. (A)** Schematic of mouse STRADα and STRADβ proteins indicating percent similarity between the two proteins. **(B)** Schematic of phylogenetic tree based on the STRAD gene from Bilaterians using Cnidaria as the outgroup with posterior probabilities indicating support for nodes (0–1, 1 being the strongest support) labeled on nodes of interest. The tree is based on MrBayes phylogenetic analysis (see Additional file 1: Figure S1). **(C)** Quantitative real-time-PCR of STRADα and STRADβ across developmental time. **(D)** Reverse transcriptase-PCR (RT-PCR) of STRADα indicates that multiple variants exist in distinct tissue types. In particular, the largest species (arrowhead) appears to be specific to brain, skeletal muscle and testes. **(E)** RT-PCR products from enriched cultures of the dominant CNS cell types. (STRADα-1 isoform – arrowhead; STRADα-4 isoform - asterisk). Cort neurons – cerebral cortex primary neurons; Interneurons – medial ganglionic eminence primary neurons, Astrocytes – primary post-natal day 1 astrocytes, Oligo – primary oligodendrocyte cultures. **(F)** RT-PCR of STRADα isoforms across developmental time in the cerebal cortex. CNS, central nervous system.

The vast networks of neural projections in the brain are essential for appropriate connectivity, but how these axons are specified during neuronal differentiation remains unclear. The regulatory mechanisms coordinating axon formation are only beginning to emerge despite the identification of key signaling molecules [1-3]. The protein kinase Liver Kinase B1 (LKB1) is a requisite component of the transduction machinery controlling axon specification both in vitro and in vivo [4,5]*.* LKB1 catalytic activity is allosterically regulated by the related pseudokinases, STRAD (STe20 Related ADapter)-alpha and STRAD-beta (STRADα and STRADβ) (Figure 1A, [6,7]). While little is known about STRADβ function, homozygous deletion within the human LYK5 (STRADα) locus (see Additional file 1: Figure S3A) results in a syndromic condition known as polyhydramnios, megalencephaly, and symptomatic epilepsy (PMSE) [8]. These patients have craniofacial dysmorphology, cognitive deficits, and intractable infantile-onset epilepsy. Given the clear impact on human health and brain development, further insights into STRAD pseudokinases are needed to clarify their contributions to nervous system development and disease.

LKB1 and either STRAD paralog form a heterotrimeric complex with either mouse protein-25 paralog, MO25α or β, proteins shown to stabilize the LKB1: STRAD dyad by binding to both proteins [6,9]. This activated heterotrimeric complex phosphorylates and activates multiple downstream kinase cascades. LKB1 substrates include the AMPK-like superfamily of kinases that are implicated in a number of physiologic roles including cell metabolism and polarity [10-13]. Interestingly, several members of this family have been shown to regulate various aspects of axon formation: SAD-A/B (also known as BRSK1/2) in the developing cerebral cortex [5,14], AMPK-α1/α2 for axonal growth under metabolic stress [15] and neuronal polarity [16] as well as NUAK1/2 regulation of axonal branching [17].

Developmentally, STRADα is expressed throughout the developing cerebral cortex, while STRADβ is found predominantly in post-migratory neurons of the cortical plate [5], suggesting that these proteins may serve distinct functions during cortical development. Many critical questions remain unaddressed regarding the roles of STRAD pseudokinases during cortical development. Importantly, it is unclear whether STRADα and STRADβ are functionally redundant during neuronal development and what distinct roles they might serve. Here, we address these questions and provide new insights into the molecular mechanisms of STRAD protein function during neuronal development.

## Results

### STRADα is the most evolutionarily conserved STRAD homolog among metazoans

Significant biochemical characterization has been conducted using vertebrate STRADs but few studies have addressed their function in vivo using genetic loss-of-function approaches. Conversely, a number of phenomenological studies, but few biochemical analyses, have been performed using invertebrate STRADs [18-21]. We conducted a phylogenetic analysis of the STRAD proteins to contextualize the relationship and functional contributions of the vertebrate paralogs. We find that metazoan STRAD proteins exhibit a high level of structural and sequence similarity within (Figure 1A) and between species (Figure 1B and see Additional file 1: Figure S1). Our analysis further suggests that a single STRAD gene duplication event occurred sometime between the origin of, and the last common ancestor for, the vertebrate lineage (Figure 1B and Additional file 1: Figure S1). The phylogenetic pattern further indicates that the vertebrate STRADα is more similar to the single invertebrate STRAD ortholog than is STRADβ. This primacy is paralleled in our loss of function studies and by previous studies demonstrating the profound effects of STRADα loss-of-function mutations in human patients [8] and by the early postnatal lethality we observe in STRADα-null, but not STRADβ-null, mice (see Additional file 1: Figure S3C, data not shown).

### A developmentally-regulated, tissue-restricted form of STRADα is expressed exclusively by neurons in the CNS

Quantitative assessment of STRAD mRNA indicates that STRADα levels decrease slightly, while STRADβ levels increase throughout cortical development (Figure 1C). Closer examination of this pattern reveals the relative contributions of STRADα splice variants (Figure 1F). A number of STRADα isoforms result from differentially utilized exons near the translation initiation site in various adult mouse tissues (Figure 1D and see Additional file 1: Figure S2A), some of which are observed in Western blots of rat tissue [22] (see Additional file 1: Figure S2A for nomenclature). Despite the fact that multiple STRADα splicing events have been described in human cell lines [23] the in vivo expression pattern of STRAD mRNA isoforms in adult tissues and during brain development has remained unknown. We characterized the expression of the STRAD variants relative to one another using reverse transcriptase PCR. Our amplicon analysis indicates that the largest STRADα variant (STRADα-1) results from the use of an alternate splice donor site and the inclusion of exon IV (see Additional file 1: Figure S2A). STRADα-1 appears predominantly in the nervous system, and to a much lesser extent in skeletal muscle and testes (arrowhead Figure 1D). Within the CNS, it is exclusive to neurons (Figure 1E) and is developmentally regulated (Figure 1F). We observe three additional splice variants previously seen and a novel fourth isoform, STRADα-7, a predicted protein in humans (GenPep EAW94290). STRADα-4 generates a larger amplicon, yet smaller predicted protein (Figure 1D, see Additional file 1: Figure S2A). This isoform has previously been reported in human tumor cell lines [23], and we detect it in most tissues (Figure 1D-F), but it is essentially absent from neurons (Figure 1E, asterisk). Similarly, human cells lines exhibit splicing in the exons encoding carboxyl regions of the protein [23], but we do not detect these splice forms in mouse tissues (see Additional file 1: Figure S2B).

Previous characterization of STRADβ indicated broad expression in adult tissues and reported a splice form that lacks exon 8 (STRADβ-2), however insufficient resolution limited prior tissue expression studies of this isoform [24,25]. Our results detect STRADβ-1 in all tissues probed and STRADβ-2 is seen in all except skeletal and cardiac muscle (see Additional file 1: Figure S2C,D). Unlike STRADα, transcriptional levels and splicing of STRADβ appear to vary less in cortical tissue and neurons across developmental time in vivo and in vitro (see Additional file 1: Figure S2E,F).

### STRADα splice forms and full-length STRADβ exhibit similar competence to drive axon formation

**Figure 2:**
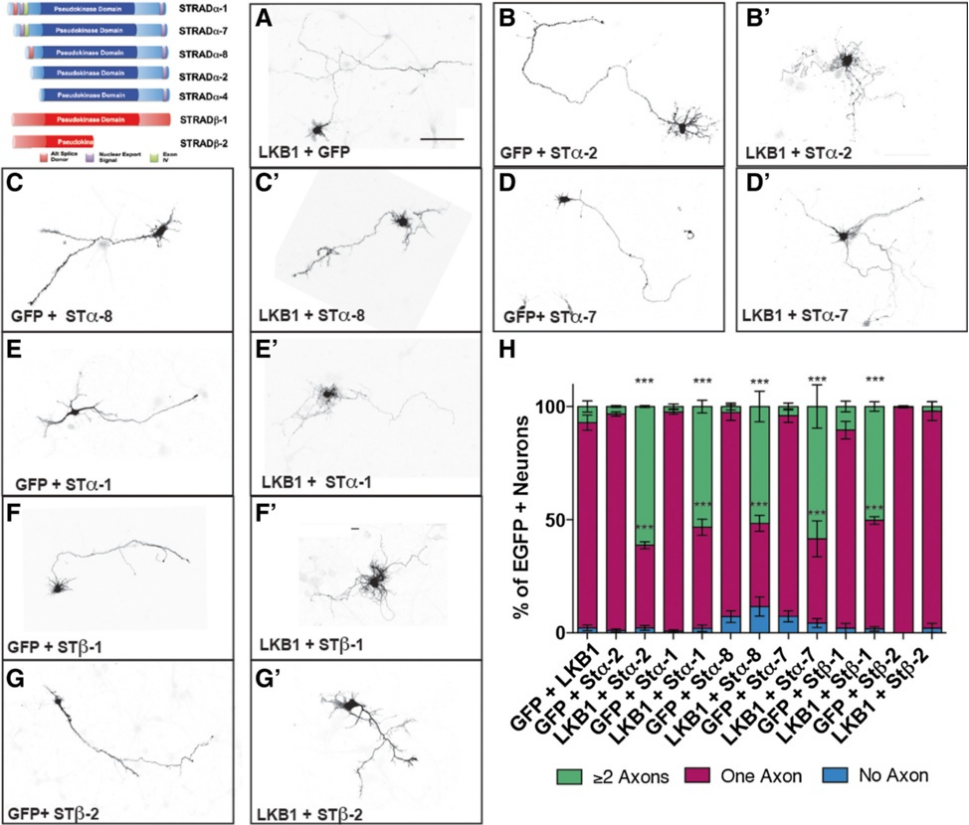
**Over-expression of STRAD isoforms leads to the initiation of multiple axons.** Multiple splice variants of each STRAD gene were tested for their ability to elicit supernumerary axons alone or with LKB1. Wild-type E15.5 cortices were electroporated with designated constructs and axon number, as determined by MAP2/Tau immuno-labeling, was quantified in GFP^+^ cortical neurons after five days in vitro. Representative neurons from each condition are shown. Neither LKB1 nor individual STRADα splice forms exhibit a significant increase in multi-axon cells **(A-G)**. When co-expressed with LKB1, all forms of STRAD are sufficient to significantly drive the formation of multiple axons **(B’-F’)** except the truncated form of STRADβ, **(G, G’)**. Scale bar = 50 μm. ***P <*0*.001 (two-way ANOVA with Bonferroni’s post-test comparing the STRAD isoform + LKB1 with the STRAD isoform + GFP). **(H)** Quantification of N = 3 to 6 independent experiments for each set of constructs. >300 GFP^+^ cells counted in each condition. ANOVA, analysis of variance; E, embryonic day; MAP2, microtubule associated protein 2.

We addressed the functional significance of each STRADα and STRADβ splice form in neurons using an *ex utero* electroporation gain-of-function approach (Figure 2). We and others have previously shown the ubiquitous STRADα-2 to be capable of driving the formation of multiple axons when expressed alone in neurons [4] or with LKB1 in neural progenitors [5]. Here, we quantified the proportion of green fluorescent protein positive (GFP^+^) cells with one or more axons after overexpression of each STRAD splice form with or without LKB1. We found that when LKB1 or each STRAD isoform was individually expressed with GFP-expressing control vector, no significant increases in axon number per neuron were observed (Figure 2A-G,H), consistent with prior observations [5].When paired with LKB1, we found that all tested STRADα splice forms are capable of eliciting multiple axons in a significant proportion of neurons, compared with the STRAD isoform + GFP-expressing vector (Figure 2B,B’-E’). In contrast, only the full-length form of STRADβ was able to induce a similar phenotype (Figure 2F’,G’) while STRADβ-2 does not significantly affect axogenesis (Figure 2G,G’).

### Genetic elimination of both STRAD genes in the cerebral cortex disrupts the formation of projection axons

**Figure 3:**
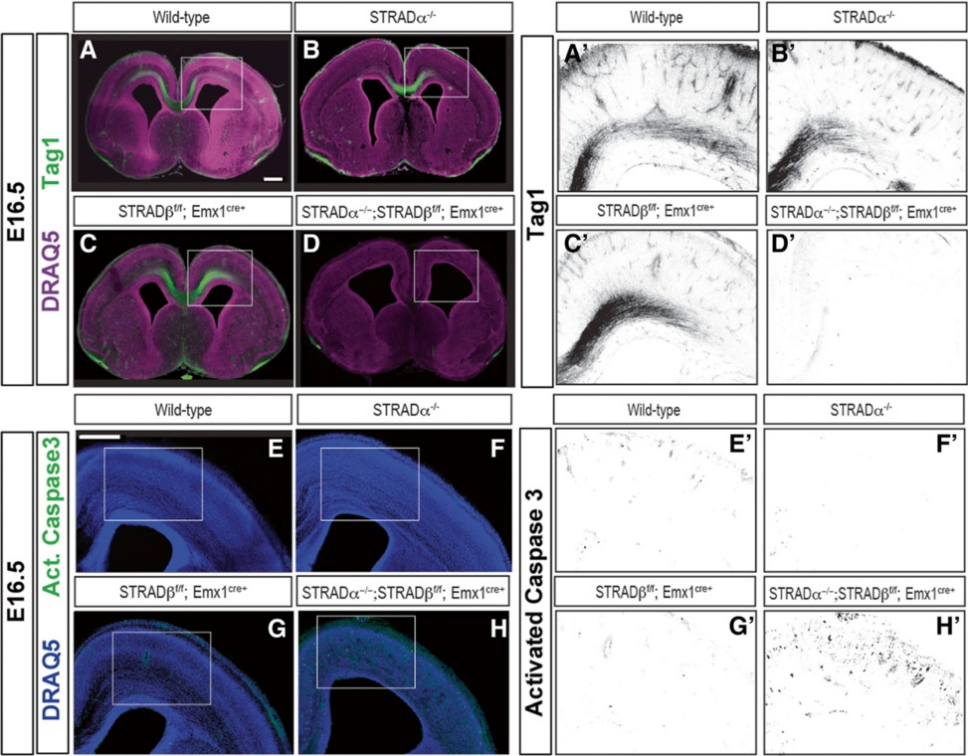
**STRADα and STRADβ are redundant in axogenesis and limiting programmed cell death.** Embryonic day 18.5 coronal sections immunolabeled for either corticofugal fibers (Tag1, green) **(A-D, A’-D’)** or activated caspase3 (Act-Casp3, green) **(E-H, E’-H’)** and a nuclear marker (DRAQ5, magenta in **A**-**D**, blue in **E**-**H**). Axons are normal in wild-type, STRADα KO and STRADβ cKO brains, but completely gone in the STRADα/STRADβ double KO brain **(A-D)**. Scale bar = 100 μm. Activated caspase3 is largely absent from wild-type, STRADα KO and STRADβ cKO brain, but drastically increased in STRADα/β double KO cortex **(E-H, E’-H’)**. Scale bar = 100 μm. Higher magnifications of cortical regions of TAG1 immunolabeling and activated caspase 3 are shown in grayscale to the right **(A’-D’ and E’-H’)**.

To address the in vivo complementation of the STRAD paralogs, we generated two novel mouse lines (a STRADα-null line and a conditional allele of STRADβ (see Additional file 1: Figure S3A)). The STRADα constitutive null animals do not produce STRADα protein (see Additional file 1: Figure S3B) and expire perinatally (see Additional file 1: Figure S3C), while the STRADβ null mice have no overt phenotypes. Histologic characterization of cerebral cortices indicated that elimination of either STRADα (Figure 3B,B’) or STRADβ (Figure 3C,C’) alone is not sufficient to disrupt axogenesis. However, deletion of both STRAD genes caused a profound loss of TAG1-positive projection axons (Figure 3D,D’), mirroring the effect observed following conditional deletion of LKB1 [5]. Similarly, neuronal polarization defects (indeterminate neurites) were observed in primary cortical cultures of STRADα/β double KO cortices compared to controls when immuno-stained for the axon/dendrite markers Tau1 and microtubule associated protein 2 (MAP2), respectively (see Additional file 1: Figure S3E-G). This indicates that either STRADα or β is sufficient to drive axogenesis during corticogenesis.

### Deletion of both STRAD paralogs results in programmed cell death in the cerebral cortex

To test whether eliminating either or both STRAD genes parallels the cortical loss of LKB1 in the context of programmed cell death, we examined activated caspase3 in cortical tissue. Immuno-staining of either the single STRADα KO (Figure 3E,E’) or STRADβ cKO (Figure 3F,F’) cortices indicates no change in apoptosis relative to wild-type control (Figure 3G,G’). In contrast, the STRADα/β double KO cortex displays substantial activated caspase3 immuno-labeling, an obvious thinning of the cortical wall, and enlarged ventricles (Figure 3H,H’), a phenotype also observed in the LKB1 cKO mice [5]. These results indicate that loss of both STRADα and STRADβ is required to phenocopy loss of LKB1, demonstrating the functional redundancy between these LKB1-activating pseudokinases, and establishing the sufficiency of STRADβ in corticogenesis.

### Loss of STRADα, but not STRADβ, reduces LKB1 stability in vivo

**Figure 4:**
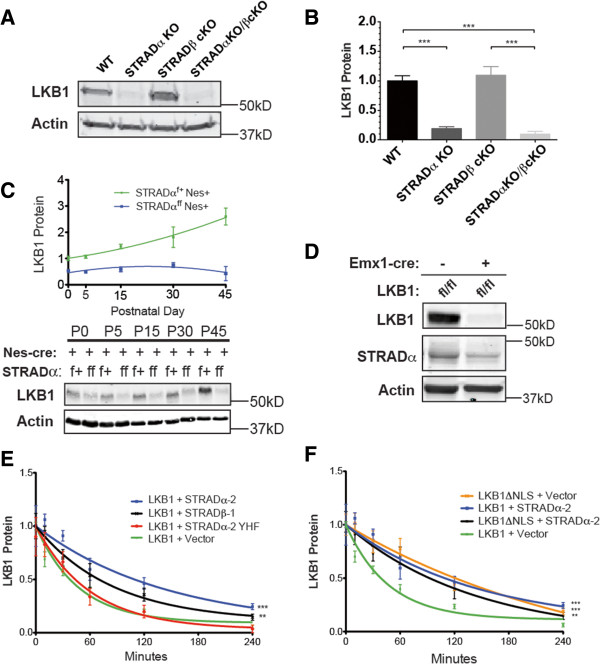
**STRADα, but not STRADβ stabilizes LKB1 protein. (A)** Representative Western blot of lysates from wild-type, STRADα KO, STRADβ cKO, or STRADα/β double KO embryonic day 16.5 (E16.5) cortex. Actin is a loading control. **(B)** Quantification of LKB1 protein levels analyzed by Western blot, normalized to wild-type cortical lysate. N ≥15 cortices from at least three litters of each genotype. There is no significant difference between columns 1 and 3 or columns 2 and 4. ***P <0.0001 using one-way ANOVA with Bonferroni’s multiple comparison test. **(C)** Western blot of Nestin-cre^+^; STRADα^f+^ or STRADα^ff^ lysates across developmental time. Quantification of LKB1 protein, normalized to postnatal day 0 (P0) STRADα^f+^; Nestin-cre^+^. N ≥3 brains of each genotype from three litters for each time-point. **(D)** Western blot of E16.5 cortical lysates showing STRADα protein is significantly reduced following conditional cortical loss of LKB1. **(E and F)** Quantification of N ≥4 independent LKB1 stability time-courses in HEK cells transfected with epitope-tagged LKB1 protein following cycloheximide-mediated translation inhibition, with three to six replicates of each condition in each experiment. **(E)** STRADα-2 significantly increases LKB1 half-life, as does STRADβ-1, to a lesser extent. **(F)** LKB1ΔNLS is as stable as LKB1 + STRADα-2 and LKB1ΔNLS + STRADα-2 is similar to LKB1ΔNLS + Vector. Error bars represent SEM. Repeated measures ANOVA with Dunnett’s multiple comparison test with LKB1 + empty vector as the control was employed. ***P <0.001, **P <0.005. ANOVA, analysis of variance; KO, knockout; SEM, standard error of the mean.

Given the similarity with LKB1 phenotypes, we tested whether endogenous LKB1 expression is affected by loss of either or both STRAD proteins in vivo*.* We find loss of STRADα leads to a significant decrease (approximately 85%) in LKB1 protein levels in the embryonic (embryonic day (E16.5) cerebral cortex (Figure 4A-B), a result mirrored in other tissues (see Additional file 1: Figure S4B). This observation is despite a significant increase in LKB1 transcription (see Additional file 1: Figure S4A), indicating that regulation of protein expression occurs predominantly post-transcriptionally. Surprisingly, this reduction in LKB1 levels impairs neither axogenesis nor cell survival (Figure 3).

In contrast, STRADβ deletion does not affect LKB1 levels, nor is the effect exacerbated by loss of both pseudokinases (Figure 4A-B). We observe a reciprocal in vivo stabilizing relationship between LKB1 and STRADα, as cortical lysates from LKB1^fl/fl^: Emx1-cre mice display a significant reduction in STRADα levels (Figure 4D), extending observations of STRADα and LKB1 stability we and others have previously made in vitro [4,26-30]. A developmental analysis using a conditional allele of STRADα (see Additional file 1: Figure S3A,D) indicates that cortical LKB1 expression is compromised by STRADα loss in all post-natal ages examined (Figure 4C), indicating that STRADα is absolutely required to achieve normal levels of LKB1. The fact that this dramatic reduction in LKB1 levels in the STRADα KO did not affect axon formation led us to explore how this alteration in protein expression impacts LKB1 phosphorylation at serine 428 (S431 in murine LKB1), a post-translational modification of LKB1 that we and Shelley et al. have shown to be critical for axogenesis [4,5]. Western blot analysis indicates that the proportion of LKB1 phosphorylated on S431 increases relative to total LKB1 in the STRADα KO, but that the total amount of pS431 is essentially unchanged (see Additional file 1: Figure S4E).

We find that HEK293 cells exhibit similar patterns of STRAD-dependent LKB1 stability (Figure 4E, see Additional file 1: Figure S4F), as observed in prior studies [4,29]. In these cells, we were able to replicate the stabilizing effects of STRADα on LKB1 by measuring protein turnover following cycloheximide-mediated protein synthesis inhibition (Figure 4E-F). These results show that STRADβ co-expression can preserve LKB1 protein levels, but to a lesser extent than STRADα. This observation likely represents sequestration of LKB1 in the cytoplasm by the predominantly cytoplasmic STRADβ. Using a previously validated LKB1-interaction mutant of STRADα-2 (YHF, Y185F/H231A/F233A) [9,31], we find that LKB1-binding by STRADα is required for this stabilizing effect (Figure 4E, see Additional file 1: Figure S4F), indicating that direct interaction mediates the stabilization of LKB1.

Previous biochemical and cell biological studies have specifically implicated STRADα in shuttling LKB1 to the cytoplasm [6,32], as STRADβ lacks key residues involved in the interaction with the nuclear export machinery [32]. We tested whether the nuclear export function of STRADα could be contributing to LKB1 stability by altering LKB1 sub-cellular localization, potentially sequestering the kinase from degradation. To do this, we examined LKB1 stability in the context of a nuclear-localization signal (NLS) mutant of LKB1 (LKB1-∆NLS) [33] (Figure 4F, see Additional file 1: Figure S5). LKB1-∆NLS demonstrates a robust stabilization when expressed alone (Figure 4F), and this increased stability is not significantly different from WT-LKB1 expressed in combination with STRADα-2, nor is the stability of LKB1-∆NLS further enhanced by co-expression of STRADα-2 (Figure 4F). Given that nuclear export of LKB1 affects its stability and that splice forms of STRADα can contain an additional nuclear export signal (NES), we tested how these isoforms (STRADα-1 and −7) impact STRAD’s ability to stabilize LKB1 and did not observe any significant differences in LKB1 stability conferred to these STRADα splice variants (see Additional file 1: Figure S4G).

## Discussion

The integral relationship between these two pseudokinases and their partner kinase is evidenced by the fact that all species bearing an LKB1 locus also contain at least one STRAD gene. Here, we provide evidence that STRADα is likely the phylogenetic ancestor of the STRAD paralogs in vertebrates, with the STRAD duplication event potentially providing selective advantage via genomic redundancy for this protein family.

Our data reveal a complex expression pattern of STRAD proteins in both developing and adult tissues and identify a new form of STRADα (STRADα-7) not previously reported. We also establish that the STRADα-1 splice form is restricted to brain, skeletal muscle and testis in the mouse. Most intriguing is our discovery that a second nuclear export sequence within the amino-terminus STRADα that has been previously demonstrated to be functional for LKB1 export [32] is developmentally regulated by splicing during brain development. While the STRADα carboxy-terminal export signal contains a phosphorylation site targeted by LKB1 [7], this N-terminal NES lacks any known post-translational modification, and may represent a less regulated form of STRADα nuclear export.

Our work and that of others demonstrates LKB1 to be a requisite component of the transduction machinery underlying axon formation [4,5] and other cell polarity hallmarks [10,34]. Acute knockdown of STRADα with small hairpin RNAs (shRNAs) leads to dysregulated mTOR signaling in the CNS [35,36], supporting the idea that STRADα is also an important regulator of nervous system function. Biochemical characterization of the LKB1: STRAD complex indicates that its formation is required for allosteric activation of LKB1 [7,22,31]. Previous neuronal loss-of-function studies have not distinguished between a requirement of LKB1 protein expression and kinase activity, but our in vivo data demonstrate that without these critical activator proteins, LKB1 is unable to elicit axon specification (Figure 3). We demonstrate that either STRAD protein is competent to drive axogenesis, and report the first in vivo physiological contribution of STRADβ.

It is important to note that previous studies evaluating STRADα function biochemically and cell biologically have often used the STRADα-1 form. Our results indicate that additional studies may be required to clarify the cellular context and specific roles of STRADα depending on which isoforms of the protein are normally expressed in a given cell type or tissue. It is possible that these amino-terminal variants of STRADα may affect recruitment of additional components to STRAD: LKB1 signaling complexes or result in alteration in localization or allosteric activation of LKB1, as previously suggested [23].

Furthermore, the increased apoptosis we observe following simultaneous inactivation of both STRAD genes provides another parallel with the LKB1 cKO [5] and suggests that allosteric activation of LKB1 plays a significant role in cell survival. This apoptosis likely results from failed axogenesis and subsequent loss of trophic support [5,17] as indicated by studies using conditional LKB1 mice and the post-mitotic cre recombinase regulated by the NEX (NeuroD6/MATH2) promoter. Whether compromised axogenesis is the sole driver triggering this cell death remains unclear, but it is possible that additional survival pathways have also been affected by LKB1 or STRADα/β loss. Future studies exploring the nature and extent of this cell death, as well as the timing of its induction relative to when cortical axons encounter their intermediate and final targets should be of great value.

Our results also indicate that STRADα uniquely plays a significant role in stabilizing LKB1 protein levels in vivo and that this stabilizing effect arises in large part from the binding and sub-cellular localization conferred upon LKB1 by STRADα. This is consistent with a previous study mapping the STRAD-MO25 interaction using carboxy-terminal deletions of STRADα [6]; however, this study did not directly implicate the deleted carboxy-terminal NES. Interestingly, the ability to stabilize LKB1 is not potentiated by STRADα isoforms containing an additional NES, leaving open the role of the splicing-regulated amino terminal export signal. The nuclear localization of LKB1 also appears to be a vertebrate specialization, as invertebrate forms of LKB1 have been shown to localize to the cytoplasm and plasma membrane [37,38] while mammalian LKB1 consistently displays a partially nuclear component (see Additional file 1: Figure S5, [32,39]. This is the first report of regulated LKB1 and STRADα stability in vivo*,* expanding upon previous work in cultured cells [4,26-30]*.* The third component of the LKB1: STRAD complex, MO25, is not affected by the loss of either protein (see Additional file 1: Figure S4C), an interesting result given previous work demonstrating that shRNA-mediated MO25 knockdown led to a reduction in LKB1 protein levels [6]. It has recently been shown that MO25 is also a critical component in regulating the Mst and OSR1/SPAK family of kinases [22] and, thus, may be stabilized through interaction with these or other partner proteins, or may not be under the same regulatory mechanism targeting STRADα or LKB1.

Efforts to understand LKB1 stability have revealed roles for heat shock proteins 70 and 90 (HSP70 and HSP90), as well as the proteasome in targeting STRADα for degradation and indicate that STRADα and HSP90 exist as competitors for LKB1 binding [26,40-42]. Here we show for the first time that a reciprocal relationship exists between STRADα and LKB1 in vivo such that LKB1 loss leads to a reduction in STRADα protein levels (Figure 4D, see Additional file 1: Figure S4D). Our observation of reciprocal stability is similar to that seen for other protein-binding partner pairs such as TSC1/2 [43] and Mdm2/SHP [44]. While it is unclear whether similar strategies exist for proteasomal avoidance in various protein pairs, this coordinated protein regulation between related and requisite components of a given signaling pathway likely represents an additional layer of coordinate regulation.

The failure of STRADβ to compensate in vivo for the loss of STRADα (Figure 4A,B) highlights the primal role of STRADα. While axon formation is not affected by STRADα deletion, additional functions within particular cell types and tissue requiring greater amounts of LKB1 may be compromised. Since PMSE patients exhibit symptoms linked to specific organ systems [8], this may reflect the lack of redundancy in LKB1 stabilization between STRADα and β. The fact that human patients often survive into early adolescence while STRADα KO mice die perinatally may also indicate additional functions or alternate developmental expression patterns for human STRADα compared to mouse STRADα.

Pseudokinases have been proposed to occupy approximately 10% of the human kinome [45] but in many cases the function of these proteins remains obscure. Our efforts assign both redundant and unique functions to two of these pseudokinases. The specific and profound nature of the effects caused by perturbing the LKB1: STRAD dyad leads to the conclusion that, as a crucial regulator of multiple signaling pathways, LKB1 signaling requires such redundancy in its activation scheme. Our evolutionary-based results illuminate a clear and meaningful phylogenetic relationship between these evolutionarily conserved pseudokinases consistent with our previous LKB1 loss of function studies. STRADα emerges as the clear primal paralog and these results present a new context in which to understand parallels between invertebrate and vertebrate functions of STRAD proteins. This analysis sets the groundwork to extend our understanding of how STRAD proteins have expanded functionalities across evolutionary time while displaying paralog-restricted specializations in vertebrate organisms. These results also demonstrate the necessary role of these proteins in the developing nervous system and add STRADs to the collection of proteins required for axogenesis.

## Conclusions

**Figure 5:**
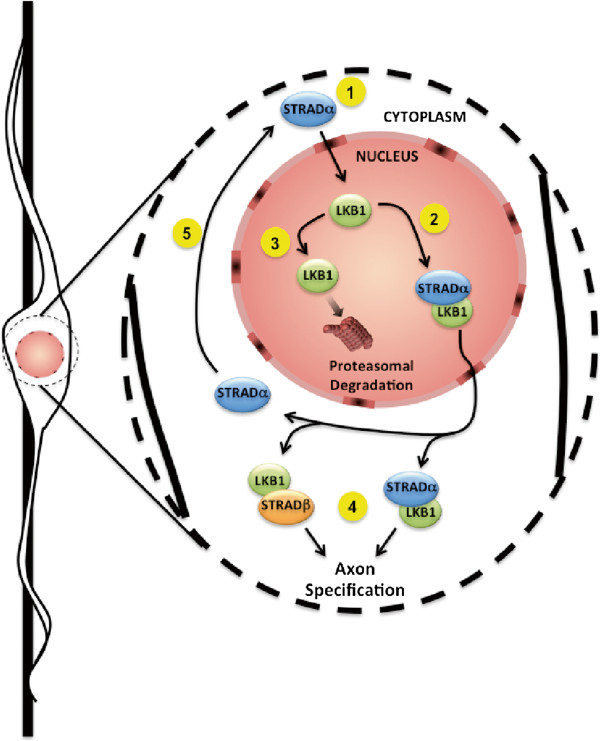
**Schematic summarizing the activation and turnover of LKB1 in a polarizing neuron as it transits along a radial glial fiber.** STRADα shuttles from the cytoplasm to the nucleus (1). At least two possible outcomes exist for nuclear LKB1 either binding to STRADα (2) or degradation via the proteasome (3). Once exported by STRADα to the cytoplasm, LKB1 can drive axogenesis (4) by either remaining bound to STRADα or by binding STRADβ. In the latter case, STRADα may return to the nucleus once again (5).

LKB1 is an important regulator of neuronal polarity and axogenesis, yet its nuanced regulation by the STRAD proteins had remained largely unexplored. Here we provide evidence of the evolutionary primacy of STRADα. We also demonstrate that messenger RNAs for the STRAD paralogs undergo a tremendous amount of tissue-restricted and cell-type specific splicing. We show that either STRADα or STRADβ is necessary and sufficient for axon formation in the developing cortex, the first report of such functional redundancy between STRADα and STRADβ (Figure 5). Most importantly, we find that only STRADα can significantly stabilize LKB1 protein in vivo and that this stabilizing effect is required for up-regulation of LKB1 expression during brain development and maturation. LKB1 stabilizes STRADα in vivo as well, indicating a reciprocal stabilizing relationship between this kinase and its regulatory partner. The normal axogenesis we observe in the absence of STRADα indicates that low levels of LKB1 are sufficient to provide the necessary signaling to permit axon specification in the developing cortex. In contrast, the perinatal lethality of these STRADα-null mice indicates a stronger sensitivity to LKB1 expression levels in other organ systems. Taken together, our data establish a previously unknown redundancy for STRADβ in axogenesis and demonstrate a unique role for STRADα in stabilizing LKB1 protein (Figure 5).

## Methods

### Phylogenetic analysis

Sequences were aligned using multiple sequence comparison by log-expectation (MUSCLE). Phylogenetic analysis was performed using MPI version of MrBayes 3.1.2 with MPICH2 installed by running in parallel on eight nodes [46,47]. Bayesian trees with posterior probabilities were constructed with mixed amino acid models, a gamma distribution for rate variation among sites and a proportion of invariable sites. MrBayes was executed with two runs (four chains for each run), four million generations of Markov Chain Monte Carlo (MCMC) analyses, with 1,000 as the sample frequency and with a temperature parameter 0.2. The number of MCMC generations assured convergence of the two runs by having a standard deviation of split frequencies less than 0.005. The posterior probability of each split was estimated by sumt with 1,000 trees discarded as burnin based on the plot of ‘generation vs. log probability’. Tree with branch lengths and posterior probabilities is shown in Additional file 1: Figure S1. Parameters were summarized by sump with 1,000 burnin, and values for the Potential Scale Reduction Factor (PSRF) were all close to 1.0 for all parameters. Scale bar represents amino acid replacements/site/unit evolutionary time.

### Animals

All mouse experiments in this study were performed using methods and protocols reviewed and approved by the Oregon Health and Science University Institutional Animal Care and Use Committee (protocol number IS00001565) or the Utrecht University Institutional Animal Care and Use Committee (protocol number HL05.1010 and governmental approval number E17) and were carried out in accordance with National Institutes of Health standards and following established guidelines of the Public Health Service. The STRADα gene trap mice were generated by random insertion of a virally-encoded splice acceptor cassette into mouse embryonic stem cells (Lexicon Genetics, see Additional file 1: Figure S3A) and STRADβ-floxed transgenics bear Cre recombinase recognition sites flanking exon 2 which encodes the start codon of the open reading frame (see Additional file 1: Figure S3A). For conditional and germ-line deletion, the STRADβ conditional line was mated to mice carry cre recombinase under the control of either the empty spiracles homeobox 1 (Emx1) or cytomegalovirus (CMV) promoter. LKB1 floxed mice are available from the National Cancer Institute Mouse Repository (strain number 01XN2)*.*

### Constructs and reagents

Total RNA from mouse tissues was extracted using Trizol reagent (Life Technologies, Grand Island, NY, USA) alone or in combination with RNeasy Mini Kit (Qiagen, Valencia, CA, USA). A total of 2 μg RNA was used as a template with Superscript VILO cDNA Synthesis Kit (Life Technologies). Alternatively, we employed a polyadenylated mRNA-derived cDNA tissue panel (Clontech, Mountain View, CA, USA). Splice forms of STRADα were amplified using Taq polymerase (Qiagen) with the following primers of the STRADα message in mouse. The sequences for 5’ segments were as follows: 5’-TGCGCTCTGACTCCTAGACC-3’ and 5’-GCTGCTCATCATCTCTGGTTT-3’. To detect 3’ splicing, primers targeting this region were: 5’-TACGGCTCTGCAAGGATCT-3’ and 5’-AGTTGGTGATGGGAGTGACTG-3’.

STRADβ was amplified using the following primers:

5’-TCTGCACCAAAATGGCTGTA-3’ and 5’-ACATCCAGTGGGCTATACGG-3’. Amplicons were purified and sub-cloned into either the TOPO-TA pCR 2.1 or pCR Blunt vector (Life Technologies) and sequenced using M13 forward or reverse primer. Quantitative RT-PCR was carried out based on the manufacturers protocol (Applied Biosystems – Life Technologies, Grand Island, NY) using total RNA extracted as described above.

### *Ex utero* electroporation and primary neuron culture

*Ex utero* electroporation was performed as described previously using a 2 μg/μL final concentration of cDNA in STRAD/LKB1 overexpression experiments (5). After electroporation, cortices were dissected, dissociated and plated on poly-D-Lysine-laminin coated coverslips (BD Biosciences, San Jose, CA, USA), cultured for multiple days in vitro in Neurobasal media supplemented with B27 supplement, penicillin-streptomycin, and Glutamax (all from Life Technologies).

### Astrocyte culture

Astrocytes were cultured according to the Banker method from P1 pups [48]. They were fed every third day with glial MEM (MEM, 20% glucose, penicillin/streptomycin, 10% heat-inactivated horse serum) – all from Life Technologies. RNA was collected once astrocytes reached confluency.

### Immunohistochemistry

Cells and tissues were fixed in 4% paraformaldehyde in phosphate-buffered saline (PBS) for 20 minutes at room temperature (cells) or overnight at 4°C (tissues) on a rotating shaker. Before immunostaining, they were blocked for one hour in 0.1% cold water fish skin gelatin/1%BSA/0.5% TritonX-100/0.01 M Tris-buffered saline (TBS) (all from Sigma St. Louis, MO), after which primary antibodies were added (see Additional file 1: Table S1) overnight at 4°C on a rotating shaker then washed 3 × 10 minutes in 1x PBS. Alexa-Fluor fluorescent secondary antibodies (Life Technologies) were applied at 1:1,000 in the same blocking solution with the addition of 5% goat serum for one hour at room temperature in the dark.

### Cycloheximide experiments

Cycloheximide (Sigma) at 100 mg/mL was used at 50 μg/mL final concentration in protein stability experiments. All protein stability experiments were carried out approximately 16 hours after transfection in Optimem media (Life Technologies). For time-courses of LKB1 degradation, cells were treated at the indicated time point relative to control, then lysed and analyzed by Western blot. The amount of LKB1 protein was normalized to actin in each sample, and compared with untreated cells from the same experiment to determine the relative amount of LKB1 protein remaining at each time point. Curves were determined by Prism5 software’s nonlinear fit of exponential decay function.

## Abbreviations

BSA: bovine serum albumin; CHX: cycloheximide; CMV: cytomegalovirus; CNS: central nervous system; E: embryonic day; EMX1: empty spiracles homeobox 1; GFP: green fluorescent protein; HSP: heat shock protein; LKB1: liver kinase B1; MAP2: microtubule associated protein 2; MO25: mouse protein 25; NEX: (NeuroD6/MATH2); NES: nuclear export signal; NLS: nuclear-localization signal; P: postnatal day; PBS: phosphate-buffered saline; PMSE: polyhydramnios, megalencephaly, and symptomatic epilepsy; shRNA: small hairpin RNA; STRAD: STe20-Related Adapter; TBS: Tris-buffered saline.

## Competing interests

The authors declare they have no competing interests.

## Authors’ contributions

BOVR conducted *ex utero* electroporation and primary neuronal and astrocyte culture, protein stability studies, western blot analysis, immuno-histochemical and -cytochemical staining. BOVR, JLS, BE, LK and KS analyzed STRAD splice variants. BOVR and AB quantified neuronal morphologies. AFB and HC generated and contributed novel mouse lines. BOVR, LK and GR generated and validated novel expression constructs. BOVR, BE, LK, APG and KS genotyped and maintained the mouse colonies. KH conducted qPCR analysis. ZZ and EG conducted the phylogenetic analysis. BOVR and APB designed the experiments. BOVR, JLS, EG and APB created a draft paper and all authors contributed critical revisions. All authors read and approved the final manuscript.

## Supplementary Material

Additional file 1: Figure S1 MrBayes tree of STRAD in metazoans. **Figure S2.** Analysis of STRAD splicing in adult tissues and during cortical development. **Figure S3.** STRAD gene structure and diagram of mutant and conditional alleles, protein expression, Punnett square analysis of STRAD-null survival, and immunocytochemistry of polarity markers in cultured neurons from the STRAD-null cerebral cortex. **Figure S4.** Analysis of LKB1 mRNA expression in the STRAD-null cortex and additional protein stability data. **Figure S5.** Subcellular localization of epitope-tagged LKB1 and STRAD variants expressed in HEK293 cells. **Table S1.** Table of antibodies used in this study. Click here for file

## References

[B1] Barnes AP Polleux F Establishment of axon-dendrite polarity in developing neurons Annu Rev Neurosci 2009 32 347 381 10.1146/annurev.neuro.31.060407.125536 19400726 PMC3170863

[B2] Polleux F Snider W Initiating and growing an axon Cold Spring Harb Perspect Biol 2010 2 a001925 20452947 10.1101/cshperspect.a001925 PMC2845204

[B3] Arimura N Kaibuchi K Neuronal polarity: from extracellular signals to intracellular mechanisms Nat Rev Neurosci 2007 8 194 205 10.1038/nrn2056 17311006

[B4] Shelly M Cancedda L Heilshorn S Sumbre G Poo MM LKB1/STRAD promotes axon initiation during neuronal polarization Cell 2007 129 565 577 10.1016/j.cell.2007.04.012 17482549

[B5] Barnes AP Lilley BN Pan YA Plummer LJ Powell AW Raines AN Sanes JR Polleux F LKB1 and SAD kinases define a pathway required for the polarization of cortical neurons Cell 2007 129 549 563 10.1016/j.cell.2007.03.025 17482548

[B6] Boudeau J Baas AF Deak M Morrice NA Kieloch A Schutkowski M Prescott AR Clevers HC Alessi DR MO25alpha/beta interact with STRADalpha/beta enhancing their ability to bind, activate and localize LKB1 in the cytoplasm EMBO J 2003 22 5102 5114 10.1093/emboj/cdg490 14517248 PMC204473

[B7] Baas AF Boudeau J Sapkota GP Smit L Medema R Morrice NA Alessi DR Clevers HC Activation of the tumour suppressor kinase LKB1 by the STE20-like pseudokinase STRAD EMBO J 2003 22 3062 3072 10.1093/emboj/cdg292 12805220 PMC162144

[B8] Puffenberger EG Strauss KA Ramsey KE Craig DW Stephan DA Robinson DL Hendrickson CL Gottlieb S Ramsay DA Siu VM Heuer GG Crino PB Morton DH Polyhydramnios, megalencephaly and symptomatic epilepsy caused by a homozygous 7-kilobase deletion in LYK5 Brain 1929–1941 2007 130 10.1093/brain/awm100 17522105

[B9] Zeqiraj E Filippi BM Goldie S Navratilova I Boudeau J Deak M Alessi DR van Aalten DMF ATP and MO25alpha regulate the conformational state of the STRADalpha pseudokinase and activation of the LKB1 tumour suppressor PLoS Biol 2009 7 e1000126 10.1371/journal.pbio.1000126 19513107 PMC2686265

[B10] Jansen M Ten Klooster JP Offerhaus GJ Clevers H LKB1 and AMPK family signaling: the intimate link between cell polarity and energy metabolism Physiol Rev 2009 89 777 798 10.1152/physrev.00026.2008 19584313

[B11] Alessi DR Sakamoto K Bayascas JR LKB1-dependent signaling pathways Annu Rev Biochem 2006 75 137 163 10.1146/annurev.biochem.75.103004.142702 16756488

[B12] Lizcano JM Göransson O Toth R Deak M Morrice NA Boudeau J Hawley SA Udd L Mäkelä TP Hardie DG Alessi DR LKB1 is a master kinase that activates 13 kinases of the AMPK subfamily, including MARK/PAR-1 EMBO J 2004 23 833 843 10.1038/sj.emboj.7600110 14976552 PMC381014

[B13] Shackelford DB Shaw RJ The LKB1-AMPK pathway: metabolism and growth control in tumour suppression Nat Rev Cancer 2009 9 563 575 10.1038/nrc2676 19629071 PMC2756045

[B14] Kishi M Pan YA Crump JG Sanes JR Mammalian SAD kinases are required for neuronal polarization Science 2005 307 929 932 10.1126/science.1107403 15705853

[B15] Williams T Courchet J Viollet B Brenman JE Polleux F AMP-activated protein kinase (AMPK) activity is not required for neuronal development but regulates axogenesis during metabolic stress Proc Natl Acad Sci U S A 2011 108 5849 5854 10.1073/pnas.1013660108 21436046 PMC3078367

[B16] Amato S Liu X Zheng B Cantley L Rakic P Man HY AMP-activated protein kinase regulates neuronal polarization by interfering with PI 3-kinase localization Science 2011 332 247 251 10.1126/science.1201678 21436401 PMC3325765

[B17] Courchet J Lewis TL Jr Lee S Courchet V Liou DY Aizawa S Polleux F Terminal axon branching is regulated by the LKB1-NUAK1 kinase pathway via presynaptic mitochondrial capture Cell 2013 153 1510 1525 10.1016/j.cell.2013.05.021 23791179 PMC3729210

[B18] Kim JSM Hung W Narbonne P Roy R Zhen M C. elegans STRAD and SAD cooperatively regulate neuronal polarity and synaptic organization Development 2010 137 93 102 10.1242/dev.041459 20023164

[B19] Narbonne P Hyenne V Li S Labbé JC Roy R Differential requirements for STRAD in LKB1-dependent functions in C. elegants Development 2010 137 661 670 10.1242/dev.042044 20110331

[B20] Chien SC Brinkmann EM Teuliere J Garriga G Caenorhabditis elegans PIG-1/MELK acts in a conserved PAR-4/LKB1 polarity pathway to promote asymmetric neuroblast divisions Genetics 2013 193 897 909 10.1534/genetics.112.148106 23267054 PMC3584005

[B21] Denning DP Hatch V Horvitz HR Programmed elimination of cells by caspase-independent cell extrusion in C. elegans Nature 2012 488 226 230 10.1038/nature11240 22801495 PMC3416925

[B22] Filippi BM de Los HP Mehellou Y Navratilova I Gourlay R Deak M Plater L Toth R Zeqiraj E Alessi DR MO25 is a master regulator of SPAK/OSR1 and MST3/MST4/YSK1 protein kinases EMBO J 2011 30 1730 1741 10.1038/emboj.2011.78 21423148 PMC3101989

[B23] Marignani PA Scott KD Bagnulo R Cannone D Ferrari E Stella A Guanti G Simone C Resta N Novel splice isoforms of STRADalpha differentially affect LKB1 activity, complex assembly and subcellular localization Cancer Biol Ther 2007 6 1627 1631 10.4161/cbt.6.10.4787 17921699

[B24] Nishigaki K Thompson D Yugawa T Rulli K Hanson C Cmarik J Gutkind JS Teramoto H Ruscetti S Identification and characterization of a novel Ste20/germinal center kinase-related kinase, polyploidy-associated protein kinase J Biol Chem 2003 278 13520 13530 10.1074/jbc.M208601200 12574163

[B25] Sanna MG da Silva CJ Luo Y Chuang B Paulson LM Nguyen B Deveraux QL Ulevitch RJ ILPIP, a novel anti-apoptotic protein that enhances XIAP-mediated activation of JNK1 and protection against apoptosis J Biol Chem 2002 277 30454 30462 10.1074/jbc.M203312200 12048196

[B26] Eggers CM Kline ER Zhong D Zhou W Marcus AI STE20-regulated kinase adaptor protein alpha (STRAD) regulates cell polarity and invasion through PAK1 signaling in LKB1 null cells J Biol Chem 2012 287 18758 18768 10.1074/jbc.M111.316422 22493453 PMC3365778

[B27] Guerreiro AS Fattet S Kulesza DW Atamer A Elsing AN Shalaby T Jackson SP Schoenwaelder SM Grotzer MA Delattre O Arcaro A A sensitized RNA interference screen identifies a novel role for the PI3K p110γ isoform in medulloblastoma cell proliferation and chemoresistance Mol Cancer Res 2011 9 925 935 10.1158/1541-7786.MCR-10-0200 21652733

[B28] Baas AF Kuipers J van der Wel NN Batlle E Koerten HK Peters PJ Clevers HC Complete polarization of single intestinal epithelial cells upon activation of LKB1 by STRAD Cell 2004 116 457 466 10.1016/S0092-8674(04)00114-X 15016379

[B29] Cheng PL Lu H Shelly M Gao H Poo MM Phosphorylation of E3 ligase Smurf1 switches its substrate preference in support of axon development Neuron 2011 69 231 243 10.1016/j.neuron.2010.12.021 21262463

[B30] Hawley SA Boudeau J Reid JL Mustard KJ Udd L Mäkelä TP Alessi DR Hardie DG Complexes between the LKB1 tumor suppressor, STRAD alpha/beta and MO25 alpha/beta are upstream kinases in the AMP-activated protein kinase cascade J Biol 2003 2 28 10.1186/1475-4924-2-28 14511394 PMC333410

[B31] Zeqiraj E Filippi BM Deak M Alessi DR van Aalten DMF Structure of the LKB1-STRAD-MO25 complex reveals an allosteric mechanism of kinase activation Science 2009 326 1707 1711 10.1126/science.1178377 19892943 PMC3518268

[B32] Dorfman J Macara IG STRADalpha regulates LKB1 localization by blocking access to importin-alpha, and by association with Crm1 and exportin-7 Mol Biol Cell 2008 19 1614 1626 10.1091/mbc.E07-05-0454 18256292 PMC2291406

[B33] Tiainen M Vaahtomeri K Ylikorkala A Mäkelä TP Growth arrest by the LKB1 tumor suppressor: induction of p21(WAF1/CIP1) Hum Mol Genet 2002 11 1497 1504 10.1093/hmg/11.13.1497 12045203

[B34] Mirouse V Billaud M The LKB1/AMPK polarity pathway FEBS Lett 2011 585 981 985 10.1016/j.febslet.2010.12.025 21185289

[B35] Orlova KA Parker WE Heuer GG Tsai V Yoon J Baybis M Fenning RS Strauss K Crino PB STRADα deficiency results in aberrant mTORC1 signaling during corticogenesis in humans and mice J Clin Invest 2010 120 1591 1602 10.1172/JCI41592 20424326 PMC2860905

[B36] Parker WE Orlova KA Parker WH Birnbaum JF Krymskaya VP Goncharov DA Baybis M Helfferich J Okochi K Strauss KA Crino PB Rapamycin prevents seizures after depletion of STRADA in a rare neurodevelopmental disorder Sci Transl Med 2013 5 182ra53 182ra53 23616120 10.1126/scitranslmed.3005271 PMC3720125

[B37] Martin SG St Johnston D A role for Drosophila LKB1 in anterior-posterior axis formation and epithelial polarity Nature 2003 421 379 384 10.1038/nature01296 12540903

[B38] Watts JL Morton DG Bestman J Kemphues KJ The C. elegans par-4 gene encodes a putative serine-threonine kinase required for establishing embryonic asymmetry Development 2000 127 1467 1475 10704392 10.1242/dev.127.7.1467

[B39] Smith DP Spicer J Smith A Swift S Ashworth A The mouse Peutz-Jeghers syndrome gene Lkb1 encodes a nuclear protein kinase Hum Mol Genet 1999 8 1479 1485 10.1093/hmg/8.8.1479 10400995

[B40] Boudeau J Deak M Lawlor MA Morrice NA Alessi DR Heat-shock protein 90 and Cdc37 interact with LKB1 and regulate its stability Biochem J 2003 370 849 857 10.1042/BJ20021813 12489981 PMC1223241

[B41] Nony P Gaude H Rossel M Fournier L Rouault JP Billaud M Stability of the Peutz-Jeghers syndrome kinase LKB1 requires its binding to the molecular chaperones Hsp90/Cdc37 Oncogene 2003 22 9165 9175 10.1038/sj.onc.1207179 14668798

[B42] Gaude H Aznar N Delay A Bres A Buchet-Poyau K Caillat C Vigouroux A Rogon C Woods A Vanacker JM Höhfeld J Perret C Meyer P Billaud M Forcet C Molecular chaperone complexes with antagonizing activities regulate stability and activity of the tumor suppressor LKB1 Oncogene 2012 31 1582 1591 10.1038/onc.2011.342 21860411

[B43] Mozaffari M Hoogeveen-Westerveld M Kwiatkowski D Sampson J Ekong R Povey S den Dunnen JT van den Ouweland A Halley D Nellist M Identification of a region required for TSC1 stability by functional analysis of TSC1 missense mutations found in individuals with tuberous sclerosis complex BMC Med Genet 2009 10 88 19747374 10.1186/1471-2350-10-88 PMC2753308

[B44] Yang Z Wang L An autoregulatory feedback loop between Mdm2 and SHP that fine tunes Mdm2 and SHP stability FEBS Lett 2012 586 1135 1140 10.1016/j.febslet.2012.03.022 22575647 PMC3350641

[B45] Boudeau J Miranda-Saavedra D Barton GJ Alessi DR Emerging roles of pseudokinases Trends Cell Biol 2006 16 443 452 10.1016/j.tcb.2006.07.003 16879967

[B46] Ronquist F Huelsenbeck JP MrBayes 3: Bayesian phylogenetic inference under mixed models Bioinformatics 2003 19 1572 1574 10.1093/bioinformatics/btg180 12912839

[B47] Altekar G Dwarkadas S Huelsenbeck JP Ronquist F Parallel Metropolis coupled Markov chain Monte Carlo for Bayesian phylogenetic inference Bioinformatics 2004 20 407 415 10.1093/bioinformatics/btg427 14960467

[B48] Kaech S Banker G Culturing hippocampal neurons Nat Protoc 2006 1 2406 2415 10.1038/nprot.2006.356 17406484

