# Supplementary material for: STRAD pseudokinases regulate axogenesis and LKB1 stability
Source: Neural Dev. 2014 Mar 4;9:5. doi: 10.1186/1749-8104-9-5 (PMC4016016; doi:10.1186/1749-8104-9-5)
Supplement: Additional file 1: Figure S1 — MrBayes tree of STRAD in metazoans. Figure S2. Analysis of STRAD splicing in adult tissues and during cortical development. Figure S3. STRAD gene structure and diagram of mutant and conditional alleles, protein expression, Punnett square analysis of STRAD-null survival, and immunocytochemistry of polarity markers in cultured neurons from the STRAD-null cerebral cortex. Figure S4. Analysis of LKB1 mRNA expression in the STRAD-null cortex and additional protein stability data. Figure S5. Subcellular localization of epitope-tagged LKB1 and STRAD variants expressed in HEK293 cells. Table S1. Table of antibodies used in this study. [file 1749-8104-9-5-S1.pdf]

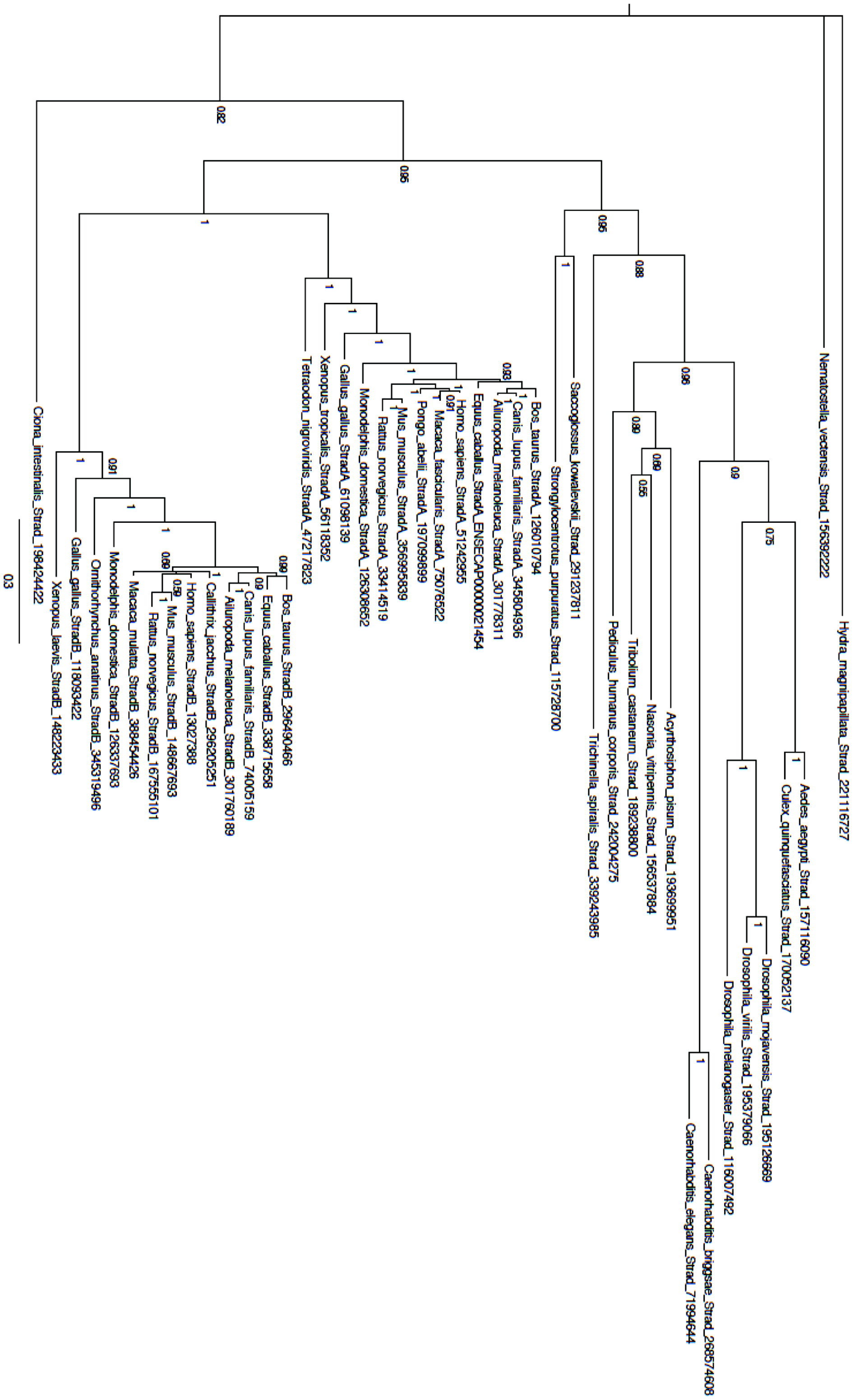

Supplementary Figure 1

MrBayes tree of STRAD gene in Metazoan using *Hydra magnipapillata* as the outgroup (46,47). The taxon name is composed of the species name, gene name (STRAD, STRADα, or STRADβ), and gene ID (NCBI GI numbers and one Ensembl protein ID for *Equus caballus* STRADa). Scale bar represents amino acid replacements/site/unit evolutionary time.

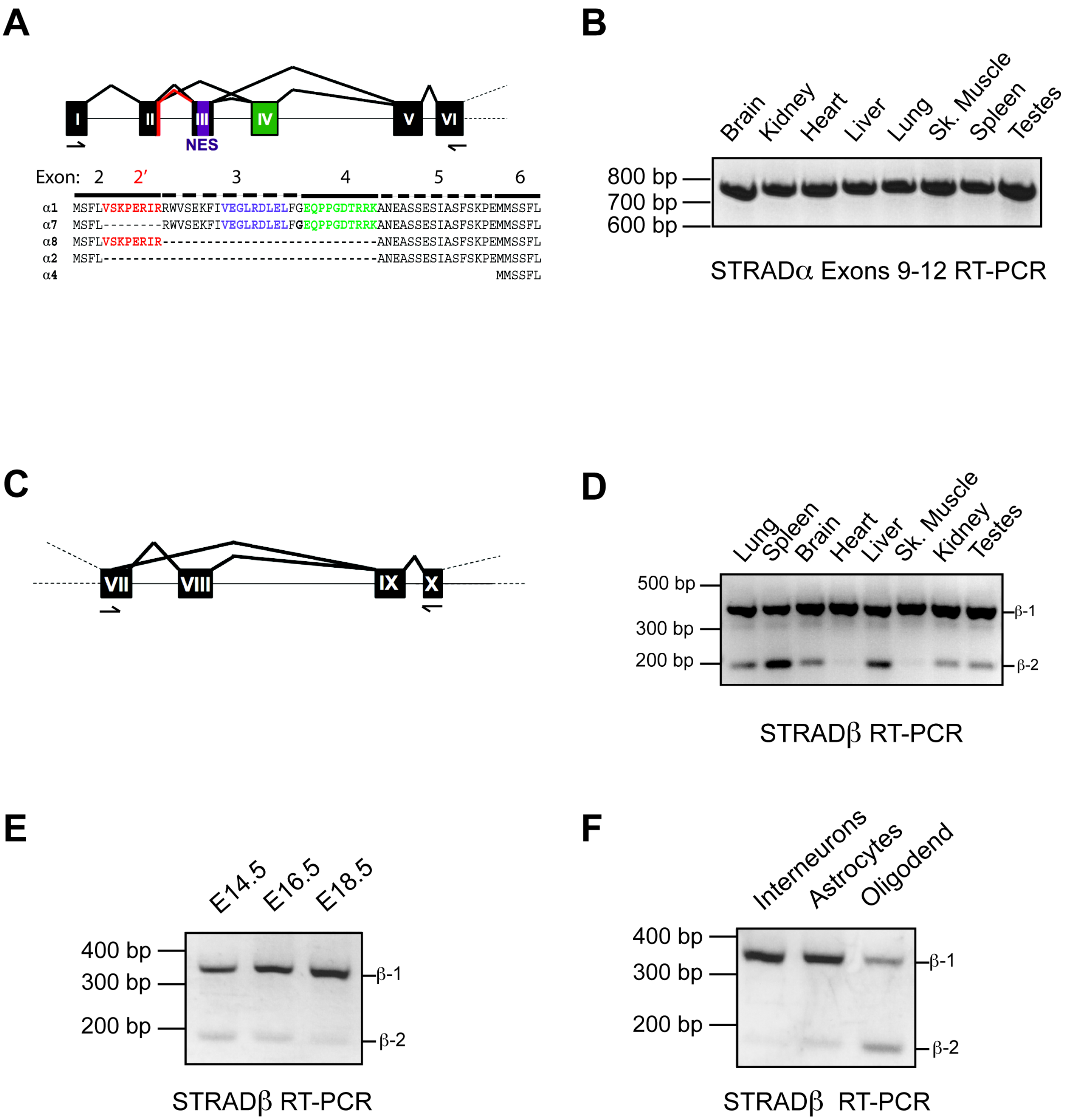

Supplementary Figure 2 - STRAD splicing is developmentally regulated (A) Amplicon sequencing indicates that at least five variants arise in the amino terminal domain of STRADα. Arrows indicate RT-PCR primer placement. (B) RT-PCR of C-terminal STRADα splicing (primers span exons 9-12) of STRAD in multiple tissues. (C) STRADβ splicing diagram in which arrows denote primers. Exon 8 is skipped in STRADβ-2 creating a premature stop codon and truncated mRNA. (D-F) STRADβ RT-PCR (D) the only reported splicing variant for STRADβ indicates that this splicing event occurs in all tissues except skeletal and cardiac muscle. (E) STRADβ splicing across developmental time in E14.5-E18.5 cortex. (F) STRADβ splice variant expression in primary cultured interneurons, astrocytes and oligodendrocytes.

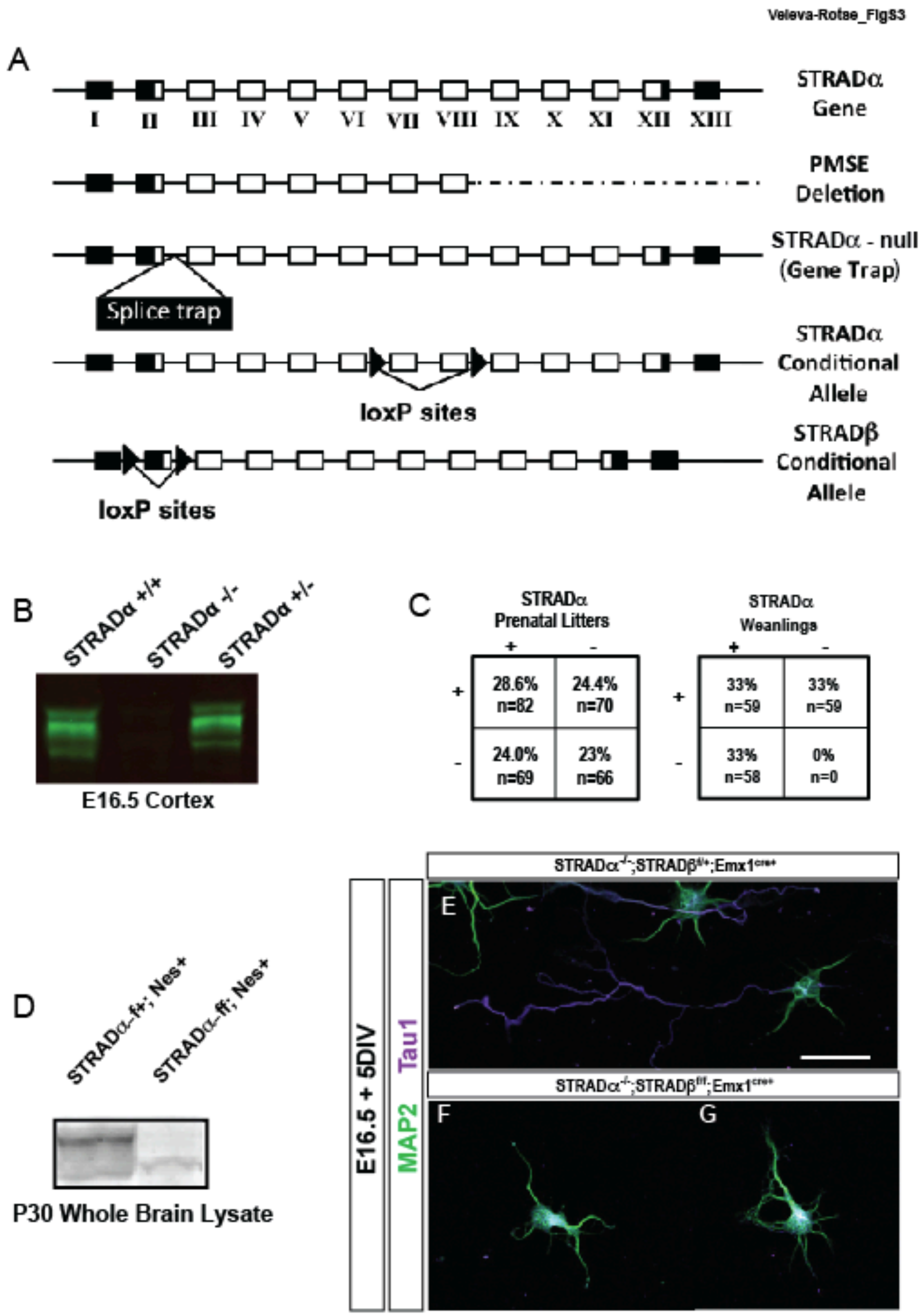

Supplemental Figure 3 - STRAD Gene Structure, Mutant and Conditional Alleles

(A) The human and mouse STRADα genes consist of 13 exons and STRADβ has 12. Schematic of the human PMSE Syndrome deletion eliminating exons 9-13. Schematic of the mouse STRADα locus disrupted via insertional mutagenesis of a splice trap construct that eliminates gene expression and the conditional allele in which exons 7 and 8 are flanked by loxP sites. Schematic of the STRADβ conditional allele in which exon 2 (which includes the start codon) is flanked by loxP sites. Black boxes indicate untranslated regions of exons and white boxes indicate the open reading frame. (B) Western blot demonstrating a loss of STRADα expression in a STRADα$^{-/-}$ E16.5 cortex, while protein level is unchanged in STRADα$^{+/-}$ cortex. (C) STRADα$^{-/-}$ mice are not viable postnatally, while almost normal Mendelian ratios exist prenatally. (D) Western blot indicating loss of STRADα protein in the P30 STRADα^{ff}; Nes-cre^{+} cortex compared with STRADα^{f+}; Nes-cre^{+} littermate. (E-G) Primary culture cortical neurons from embryonic day 16.5 mouse cortex grown for 5 days *in vitro* and immunolabeled for MAP2/Tau. Indeterminate neurites are seen in (F-G) STRADα$^{-/-}$;STRADβ$^{fl/fl}$;Emx1-cre^{+} neurons but not in (E) STRADα$^{+/-}$;STRADβ$^{fl/fl}$;Emx1-cre^{+}. Scale bar=20um.

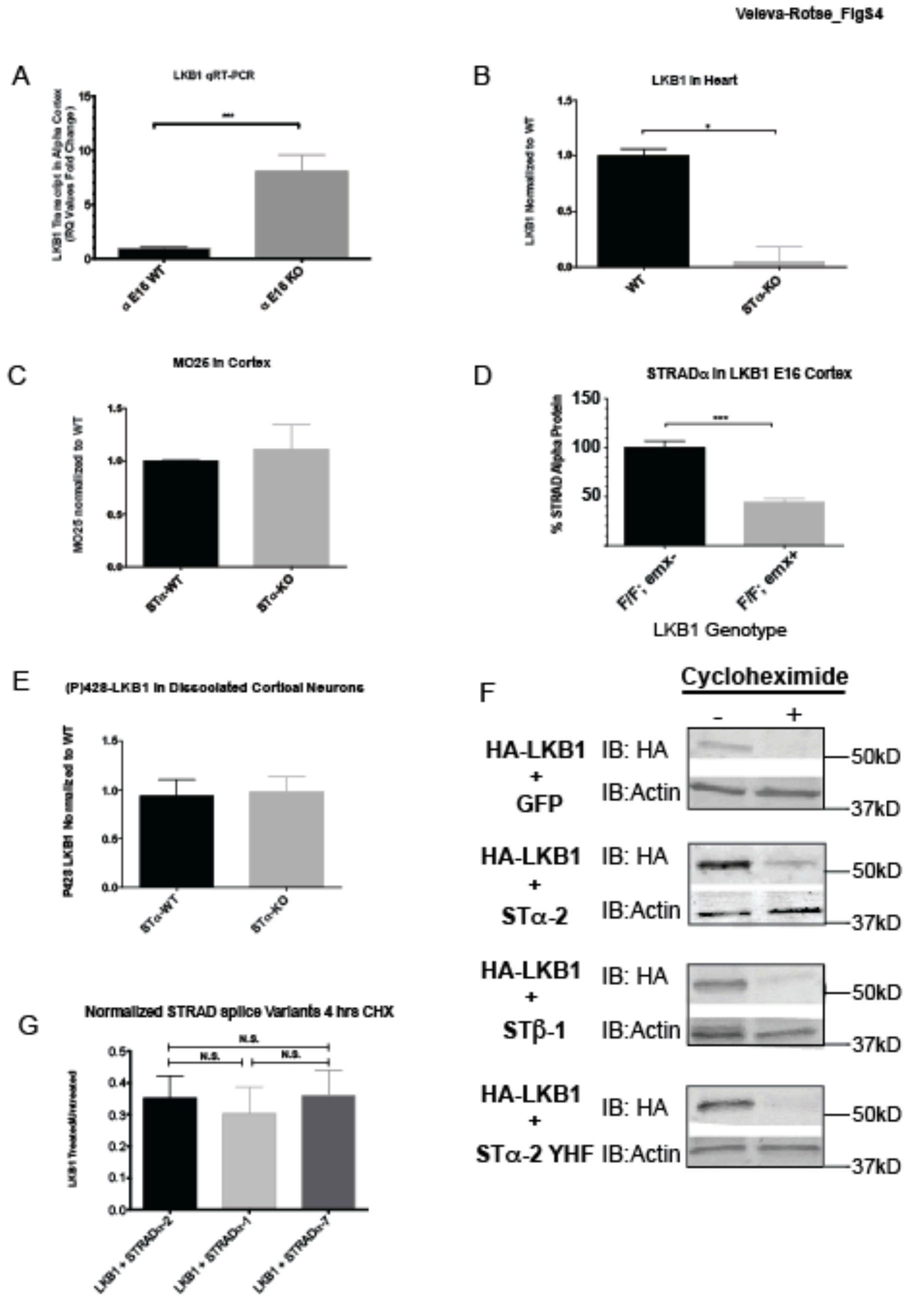

Supplemental Figure 4 – LKB1 Stability

(A) Up-regulation of LKB1 mRNA in $STRAD^{-/-}$ (KO) E16.5 cortex relative to $STRAD^{+/+}$(WT) as assessed by qRT-PCR. WT N=3, KO N=4 from 2 different litters. Quantification of Western blot replicates: (B) Significant destabilization of LKB1 protein in STRADα KO E16.5 embryonic hearts. WT N=2, KO N=4 (C) MO25 levels are unchanged in STRADα KO E16.5 cortex compared with WT. WT N=2, KO N=4. (D) STRADα protein is destabilized in $LKB1^{ff}$; $Emx1\text{-}Cre^{+}$ E16.5 cortex compared with littermate controls. N ≥ 10 cortices of each genotype from at least 3 litters. (A-D) Error bars represent SEM; *p<0.05, ***p<0.001 by Student's T-test. (E) Quantification of western blot analysis of the levels of LKB1 phosphorylated at serine 428/431 in primary cultures derived from either control or STRADα-null mice. (F) HEK293 cells were transfected with HA-tagged LKB1 and STRAD constructs. Representative Western blot anti-HA of untreated and 4hrs 50μg/mL cycloheximide treated cells, with actin as a loading control. (G) Quantification and comparison of LKB1 stability following 50 μg/ml cycloheximide treatment when co-transfected with STRADα splice variants STRADα-2, -1 or -7.

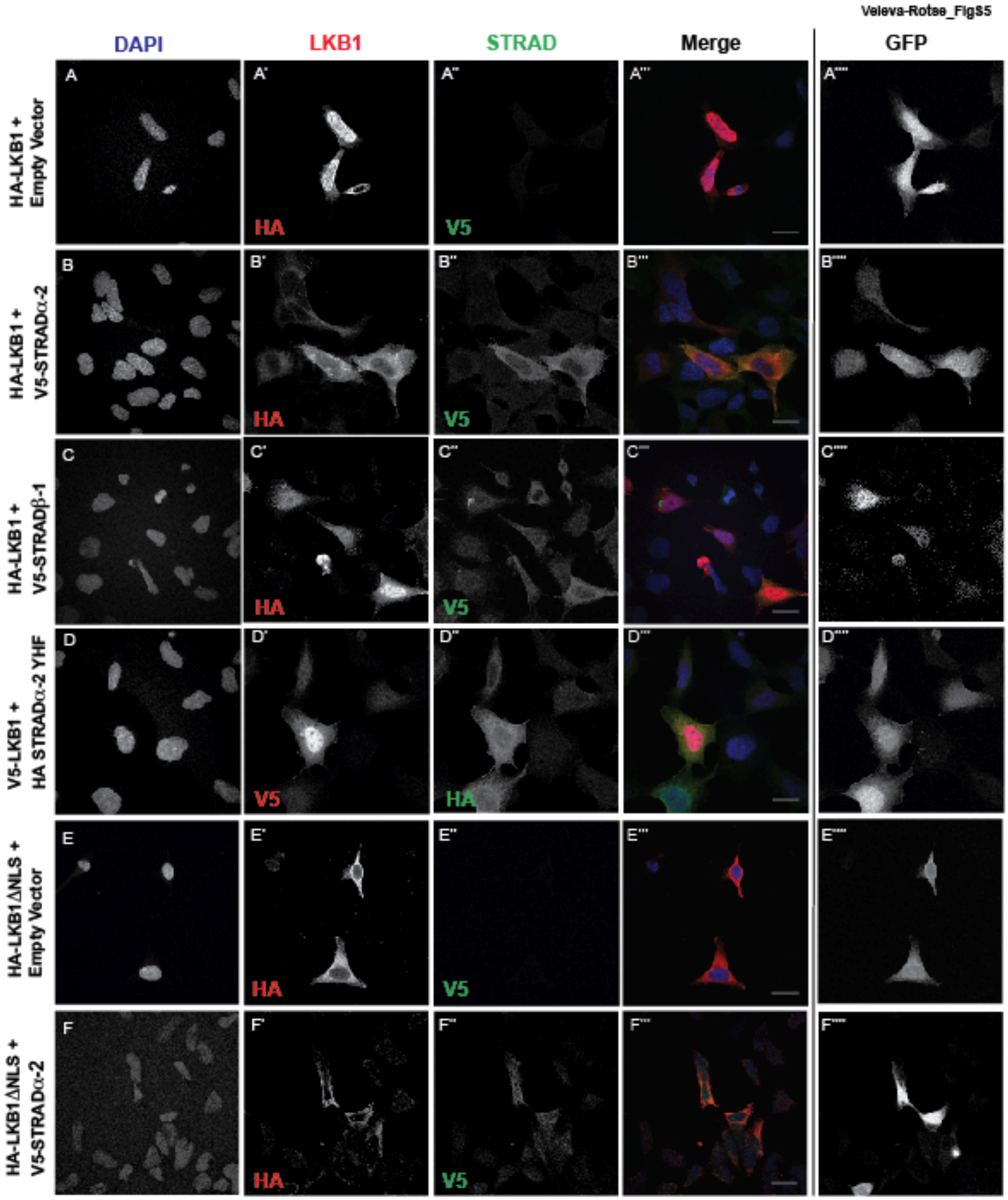

Supplemental Figure 5 – Subcellular localization of epitope-tagged LKB1 and STRAD Variants

Verification of subcellular localization for epitope-tagged LKB1 and STRAD constructs transiently transfected in HEK293 cells. (A-A'''') LKB1+GFP-expressing empty vector, (B-B'''') LKB1+STRADα-2. (C-C'''') LKB1+STRADβ-1, (D-D'''') The LKB1+STRADα-2 YHF (LKB1 binding mutant), (E-E'''') LKB1ΔNLS+GFP-expressing empty vector (F-F'''') LKB1ΔNLS+STRADα-2.

Table S1

Target Protein	Species	Company	Concentration Used
Actin	Mouse	Millipore	1:10,000 (WB)
Cleaved Caspase3	Rabbit	Cell Signaling	1:1000 (IF)
GFP	Chicken	Aves	1:1000 (IF)
LKB1 D60C5	Rabbit	Cell Signaling	1:1000 (WB)
MAP2	Rabbit	Chemicon	1:10,000 (IF)
MAP2	Chicken	Covance	1:10,000 (IF)
MO25α	Rabbit	Cell Signaling	1:1000 (WB)
STRADα	Rabbit	Sigma	1:1,000 (WB)
Tag1	Mouse IgM	DSHB	1:50 (IF)
Tau-1 Clone PC1C6	Mouse	Millipore	1:1,000 (IF)
Tubulin (βIII)	Mouse	BD	1:1000 (IF)

Table of antibodies used.
